# Electric Energy Storage Effect in Hydrated ZrO_2_-Nanostructured System

**DOI:** 10.3390/nano12111783

**Published:** 2022-05-24

**Authors:** Alexander S. Doroshkevich, Andriy I. Lyubchyk, Boris L. Oksengendler, Tatyana Yu. Zelenyak, Nurbol O. Appazov, Andriy K. Kirillov, Tatyana A. Vasilenko, Alisa A. Tatarinova, Oksana O. Gorban, Viktor I. Bodnarchuk, Nadejda N. Nikiforova, Maria Balasoiu, Diana M. Mardare, Carmen Mita, Dorin Luca, Matlab N. Mirzayev, Asif A. Nabiyev, Evgeni P. Popov, Anca Stanculescu, Tatyana E. Konstantinova, Yulia V. Aleksiayenak

**Affiliations:** 1 Donetsk Institute for Physics and Engineering Named after O.O. Galkin, 03028 Kyiv, Ukraine; oxanag1@ukr.net (O.O.G.); alta7@ukr.net (T.E.K.); 2 Joint Institute for Nuclear Research, Joliot-Curie 6, Dubna, 141980 Moscow, Russia; tatyana.zelenyak@bk.ru (T.Y.Z.); kirillov1953@inbox.ru (A.K.K.); s191983@stud.spmi.ru (A.A.T.); bodnarch@nf.jinr.ru (V.I.B.); masha.balasoiu@gmail.com (M.B.); matlab@jinr.ru (M.N.M.); asifnebi@gmail.com (A.A.N.); epetropov@gmail.com (E.P.P.); beataa@gmail.com (Y.V.A.); 3 Nanotechcenter LLC, Krzhizhanovsky str. 3, 03680 Kyiv, Ukraine; p6193@ulusofona.pt; 4 Research Centre in Industrial Engineering Management and Sustainability, Lusófona University, Campo Grande, 376, 1749-024 Lisbon, Portugal; 5 Ion-Plasma and Laser Technologies Institute after U. Arifov, Tashkent 100125, Uzbekistan; oksengendlerbl@yandex.ru (B.L.O.); 99_959@bk.ru (N.N.N.); 6 I. Zhakhaev Kazakh Scientific Research Institute of Rice Growing, Abay Avenue 25B, Kyzylorda 120008, Kazakhstan; nurasar.82@mail.ru; 7 Laboratory of Engineering, Korkyt Ata Kyzylorda University, Ayteke bi Street, 29A, Kyzylorda 120014, Kazakhstan; 8 Saint-Petersburg Mining University, 199106 St. Petersburg, Russia; tvasilenko@mail.ru; 9 Horia Hulubei National Institute for R&D in Physics and Nuclear Engineering (IFIN-HH), Str. Reactorului no. 30, P.O. Box MG-6, 077125 Magurele, Romania; 10 Faculty of Physics, “Alexandru Ioan Cuza” University of Iasi, Bld. Carol I, No. 11, 700506 Iasi, Romania; dianam@uaic.ro; 11 Faculty of Chemistry, “Alexandru Ioan Cuza” University of Iasi, Bld. Carol I, No. 11, 700506 Iasi, Romania; cmita@uaic.ro; 12 Faculty of Materials Science and Engineering, “Gheorghe Asachi” Technical University of Iasi (TUIASI), bd. Dimitrie Mangeron, nr. 41, 700050 Iasi, Romania; dluca@tuiasi.ro; 13 Institute of Radiation Problems, Azerbaijan National Academy of Sciences, Baku AZ1143, Azerbaijan; 14 Institute of Solid-State Physics, Bulgarian Academy of Sciences, 1784 Sofia, Bulgaria; 15 Institute for Nuclear Research and Nuclear Energy, Bulgarian Academy of Sciences, 1784 Sofia, Bulgaria; 16 Optical Processes in Nanostructured Materials Laboratory, National Institute of Materials Physics, 405A Atomistilor Street, P.O. Box MG-7, 077125 Magurele, Romania; sanca@infim.ro

**Keywords:** zirconium oxide, powder nanotechnologies, nano-ionic capacitors, nanoelectronics, dimensional effects

## Abstract

The dimensional effect of electric charge storage with a density of up to 270 μF/g by the hydrated ZrO_2_-nanoparticles system was determined. It was found that the place of localization of different charge carriers is the generalized heterophase boundary-nanoparticles surface. The supposed mechanism of the effect was investigated using the theory of dispersed systems, the band theory, and the theory of contact phenomena in semiconductors, which consists of the formation of localized electronic states in the nanoparticle material due to donor–acceptor interaction with the adsorption ionic atmosphere. The effect is relevant for modern nanoelectronics, microsystem technology, and printed electronics because it allows overcoming the basic physical restrictions on the size, temperature, and operation frequency of the device, caused by leakage currents.

## 1. Introduction

The development of modern solid-state electronics, such as wireless networks of sensors, autonomous objects of nano- and microsystem technology such as “smart dust” (~1 mm^−3^) and “nanomorphic cell” (~1 mm^−6^) [1,2], RF microchips identification, terahertz microsystems for bio-identification, and military applications [3,4] is limited due to the lack of submicroscopic capacitors with a high capacitance density (>100 μF/g) capable of operating frequencies up to 109 Hz [5]. In modern processors, where the temperature reaches 85–100 °C, electronics for deep drilling installations (temperature above 150 °C), instrument compartments of spacecraft, there is an acute problem of overheating of electronics (at 150 °C, the service life of ferroelectric capacitors is several hours). Thus, an urgent scientific and technological task is the development of electrical capacitors with high and ultra-high capacitance density, capable of operating at temperatures above 150 °C at frequencies up to 1 GHz and higher.

The type of solid-state storage devices for nano-ionic pulses of a planar design is the main concept for the development of such devices today. This concept is based on the localization of charge at the interface between materials with different types of conductivity: electronic and ionic. To achieve high surface potentials [3,6,7], work is underway to (1) form an atomically clean and sharp contact at the junction between conductors with ionic and electronic types of conductivity; (2) ensure minimal disordering of the structure in the superionic conductor layer adjacent to the electronic conductor at the corresponding coherent boundaries; (3) provide a certain combination and mutual arrangement of the symmetry elements of the heterojunctions and the symmetry elements of the fast ion transport channels in the structure of the superionic conductor. The design of these devices allows a satisfactory level of capacitance in the operating frequency and temperature range up to 1 GHz and 180 °C (at 1 MHz), correspondently [8,9]. However, the level of miniaturization of equipment required by nanoelectronics and microsystems is limited by a “parasitic” size effect, called the “effect of tunneling charge exchange of surface electronic states in a dielectric layer of critical thickness” [5]. When the size of this nanoion device decreases below the critical size, a leakage current occurs in the ion conductor, significantly reducing the capacitance of the device. Thus, the tunneling effect imposes physical constraints on sizes, operating temperature, and amount of specific capacitance of nano-ionic pulsed solid-state storage devices, and the technical specifications of devices in which they are used.

The works [10,11] indicate the presence of electron charge carriers associated with adsorbates in the near-surface region of nanoparticles, which determine the surface capacitance. With small particle sizes, this capacitance can be high, and the operating temperature and frequency during electrolyte selection can reach large values.

The purpose of this work was to study the electrical properties of nanoscale particles of a wide-band dielectric as part of concentrated dispersed systems with a humid-gas dispersion medium and to develop a functional medium for creating submicroscopic nanoion storage devices of ultra-high capacitance for nanoelectronics and microsystem technology alternative planar design.

## 2. Materials and Methods

The samples of investigated concentrated nanopowder oxide dispersed systems (NODS) in the form of tablets (thickness 2 mm and diameter 18 mm) were obtained by high hydrostatic pressure (HHP 500 MPa) from monodispersed powders of ZrO_2_-3 mol%Y_2_O_3_ obtained by the co-precipitation method [12]. The particle size in the powders was set by thermal annealing in the temperature range above 400 °C.

To avoid significant discrepancies in the physical and chemical parameters of the samples, their structural state was unified by special physical processing before each experiment. Before the measurement, all samples were dried in a drying cabinet for 60 min at a temperature of 120 °C, and then in a climate chamber, they were saturated with moisture in an atmosphere with a relative humidity of η = 70%, for 180 min.

By changing the humidity of the atmosphere, in the climate chamber, the relative amount of moisture in the samples was varied $\Delta m=\frac{m-m_{0}}{m}$.

The lowest moisture value in this work, Δ*m* = 1.7 wt%, was achieved at atmospheric humidity η = 25%, and the highest Δ*m* = 6.6 wt%—at η = 85%.

To measure the electrical properties, carbon contacts were mechanically deposited at the ends of the samples.

**Figure 1:**
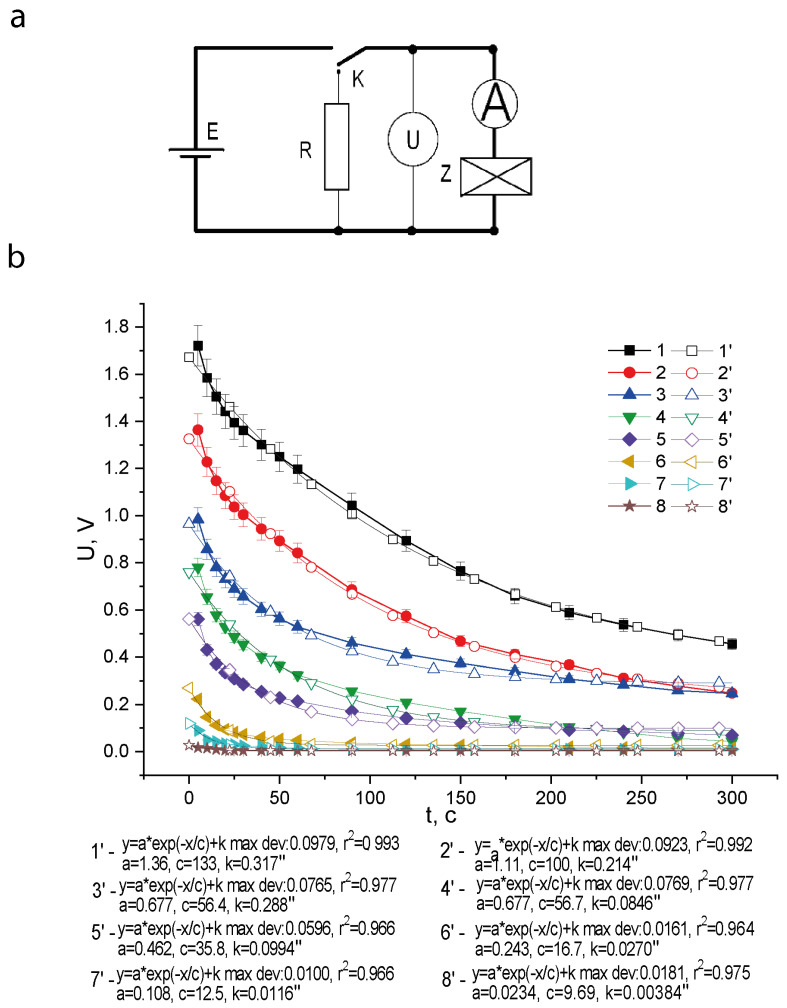
Experimental electrical circuit (**a**) and obtained family of discharge characteristics of images (**b**) for nanoparticles of the initial powder *d*: 7.5 nm (1), 9 nm (2), 12 nm (3), 18 nm (4), 23 nm (5), 50 nm (6), 67 nm (7), 96 nm (8). Fit curves are numbered: 1’, 2’, 3’, 4’, 5’, 6’, 7’, 8’. The electrical circuit for obtaining (**a**), where Z is the sample; R—electrical load, K—switch, E—power supply. Charge field *E* = 5 V/mm, charge time *t* = 10 min, atmospheric humidity η = 85%.

The charge–discharge characteristics were studied by voltammetry using a precision multimeter of the AM1199 type (Figure 1). The dependences of the load voltage on the discharge time of the sample were recorded. The electrical diagram of the measuring circuit is shown in Figure 1a. The values of the external field and the electrical load were: *E_ex_* = 5 V/mm and *R*_0_ = 1 MΩ, correspondently.

The exposure of the samples to an electric field was carried out directly in a climatic chamber at normal: atmospheric pressure (762 Hg), and temperature +22 °C for 10 min. Depending on the experimental conditions, the air humidity in the chamber varied from 25% to 85%.

The analysis of the physical and chemical properties of the objects of study before and after exposure to an external electric field was carried out using the methods of BET (Brunauer–Emmett–Teller), nuclear magnetic resonance (NMR), gravimetric (GA), and thermogravimetric (TGA) analysis.

The method for determining the thermal adsorption–desorption of nitrogen molecules by porous objects (BET, Sorbi 4.1 type device) was used for qualitative analysis of the chemical activity of the sample surface.

NMR and TGA studies were carried out by a wide-line spin-echo spectrometer operating at a frequency of 20 MHz (the resonant frequency for ^1^H hydrogen nuclei) and two ADS50-type moisture meters.

The spin-echo NMR spectrometer was used to determine the spin-lattice (*T*_1_) and spin-spin (*T*_2_) relaxation times before and after the samples were exposed to an electric field. The hitch was 0.9. When calculating the spin-lattice relaxation time *T*_1_, a two-pulse technique was used, in which a change in the pulse repetition frequency *f* is provided [13].

Gravimetric measurements were carried out in triplicate on an AXIS AN-200 analytical balance. During exposure to an electric field, the sample was removed from the working cell and weighed separately from the contacts.

Four identical samples were used for the TGA studies. Two of them (“EXPERIMENT”) were exposed to an electric field, and the second pair (“CONTROL”) was used as a control group. Moisture desorption was carried out at 90 °C and 70 °C. The control and experimental samples were simultaneously dried in pairs on two separate scales-moisture meters. The rate *k* and the activation energy *E_a_* of water desorption from the surface of nanoparticles were determined.

The spatial structural organization of the samples was studied by scanning (SEM, JSM640LV) and transmission (TEM, JEM 200A) electron microscopy [14]. The crystal structure of the defects was determined from electronographic images obtained using TEM. Detailed studies of the structure of the nanopowders used are given in [15]. The electrical properties of the samples were studied by electrochemical impedance spectroscopy (EIS).

EIS methods were used to determine the spatial distribution of phases and to characterize the electrical properties of the materials that make up the samples under study. A precision virtual meter-analyzer of impedance parameters of the 2B-1 type was used [16,17]. The amplitude of the alternating exciting signal is 50 mV, the frequency range is 50 Hz–1 MHz, and the measurement time of one point is 5 s. The calculation of the impedance parameters was carried out using a computer program [18,19]. The methodology of EIS studies of the NODS in the author’s version is described in detail in [11,15,20,21].

## 3. Results

Figure 1b shows a family of discharge curves of samples and the corresponding curves obtained as a result of their fitting. It can be seen that the exposure of samples in an electric field of the order *E* = 5 V/mm for *t* = 10 minutes at a humidity of η = 85% leads to the induction of a potential difference *U* up to 2 V on their electrodes (Figure 1b, Table 1).

The maximum values of the residual electric field corresponding to the voltage U_0_ at the electrodes are observed at a minimum particle size *d* of about 7.5 nm. The effect is practically absent at *d* ≥ 96 nm.

The discharge curves (Figure 1b) are exponential and can be fitted by the equation [22,23].

(1) $$U=U_{0}\exp\left(-\frac{t}{\tau_{dis}}\right),$$

where *U*_0_ is the value of the voltage between the electrodes at the initial moment *t* = 0.0 s; *R* = 1 MΩ—discharge circuit resistance; *τ_dis_ =* R·C—the discharge time.

The coefficients *a*, *c*, *k* are approximated by a theoretical dependence for an RC circuit. The accuracy of the approximation is characterized by the determination parameter R^2^. The parameter c is identical with the relaxation time *τ_dis_*. It can be seen that the approximation by one exponent satisfactorily describes the curve for a qualitative assessment of the size factored effect.

**Figure 2:**
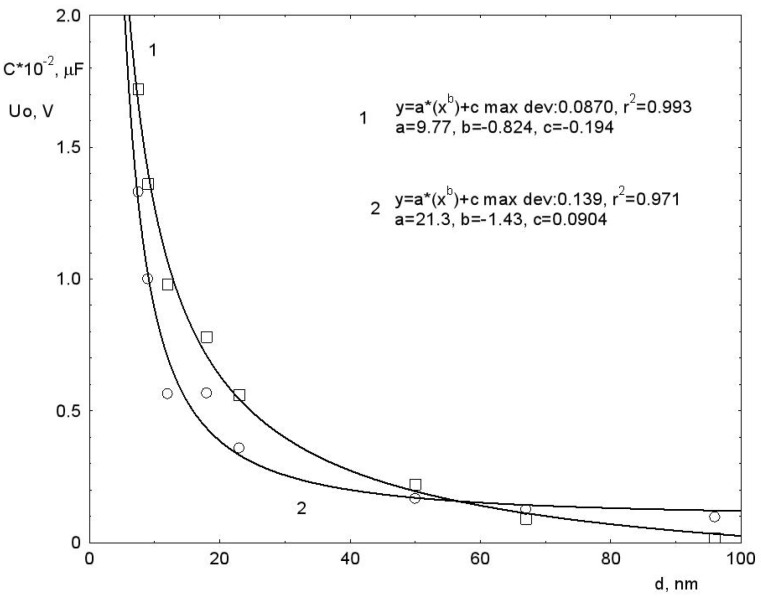
Dependences of the capacitance *C* and voltage *U*_0_ at the electrodes of the samples on the size of its constituent nanoparticles (η = 85%). The marked points show the experimental results. Curves without symbols correspond to calculated curves.

The *C* values for the curves in Figure 1b are given in Table 1 and in graphical form—in Figure 2.

It can be seen from Figure 2, that for *C* there is the same dimensional dependence as for *U*_0_. The capacitance value decreases rapidly with increasing particle size. The maximum capacitance *C_max_* takes place at *d* = 7.5 nm (Figure 1b curve 1) and is *C_max_* = 1.87 × 10^−4^ F/g. The minimum capacitance *C_min_* = 19.2 occurs at *d* = 96 nm.

Thus, it follows from Figure 2 and Table 1 that the studied effect of accumulation of an electric charge by the system has a dimensional character.

It was also found that the dividing on the parts of the sample does not lead to the loss of the state of “polarization”, and the capacitance *C* proportionally depends on the moisture content in the sample (Table 2).

Δm was determined by the formula:

(2) $$\Delta m=\frac{m-m_{0}}{m}$$

In particular, the electrical capacitance of the sample decreases monotonically by a factor of 34.5 as water is desorbed from it from Δ*m* = 6.6 wt%.

**Figure 3:**
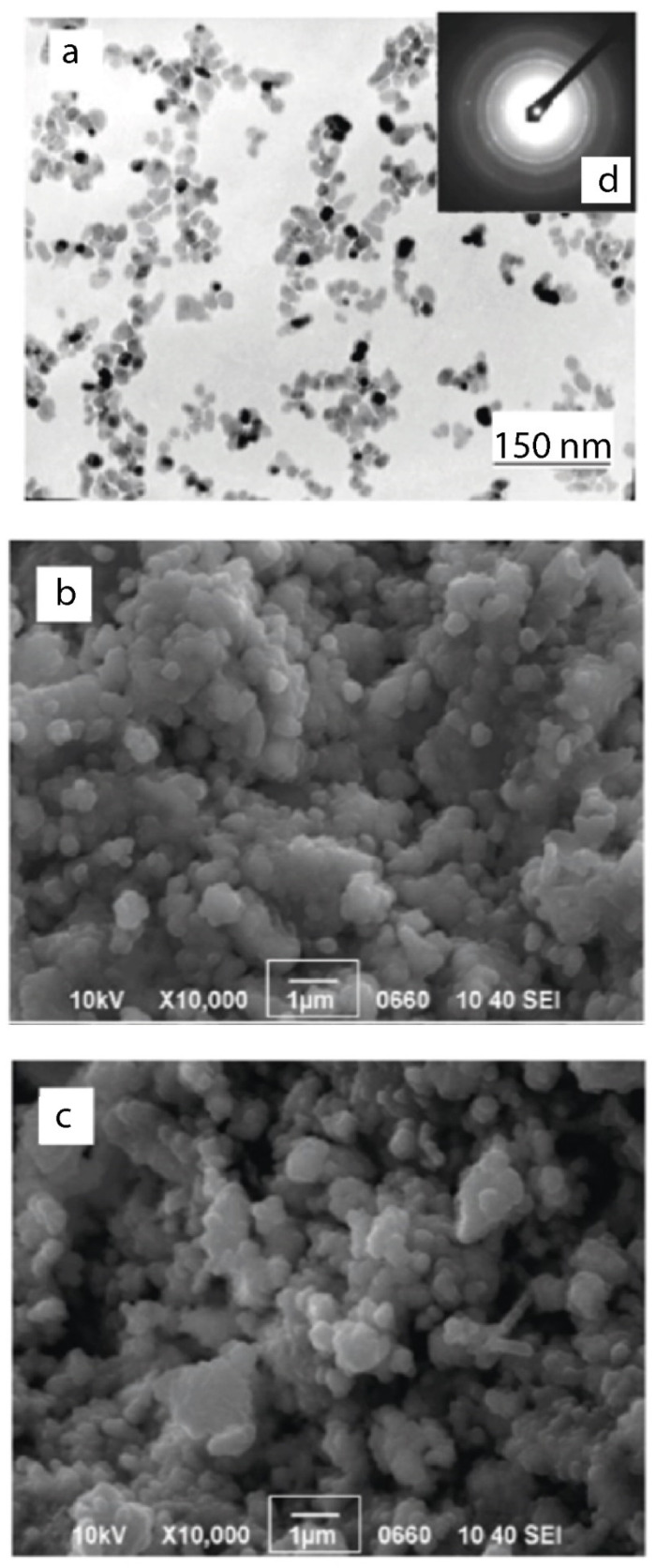
TEM image of a nanopowder (particle size *d* = 7.5 nm) of the composition ZrO_2_-3 mol% Y_2_O_3_, used to produce samples (**a**). Sample fracture topology before (**b**) and after (**c**) exposure to an electric field (*E* = 5 V/mm, *t* = 10 min), and an electrogram showing the crystal structure of nanopowders (**d**).

The images in Figure 3, obtained by electron microscopy methods, reflect the characteristic features of the morphology of the powders and the spatial structure of the objects of study.

### 3.1. Electrochemical Impedance Spectroscopy. The Electrical Structure of the Samples

**Figure 4:**
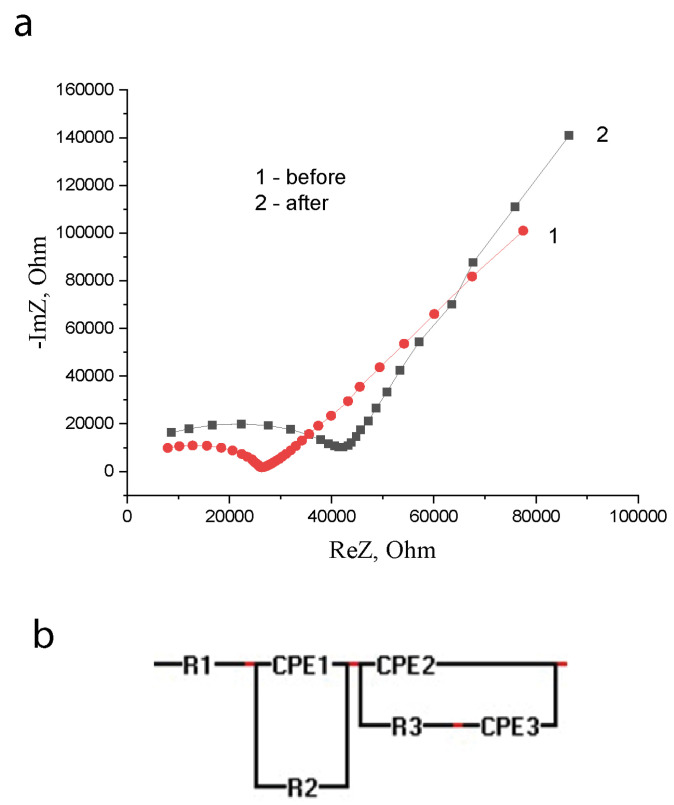
Typical hodographs of samples with a particle size *d* = 7.5 nm before and after exposure to an electric field *E* = 5 V/mm for *t* = 10 min (**a**). The equivalent electrical circuit of the object (**b**).

Figure 4 shows typical hodographs of the samples before and after exposure to an electric field.

The given spectra contain information on the electrical structure of objects and allow us to determine the effect of an electric field on them.

The presence of several elements in the EIS spectra (Figure 4a,b) indicates that the sample is spatially inhomogeneous in electrical properties.

This means that the sample contains several physical phases with different electrical properties. These phases can be spatially distributed in the sample as desired. The phases give a similar electrical response in a specific frequency range to the excitation signal of the spectrometer. And their response time to an external RF excitation signal can be significantly different.

**Figure 5:**
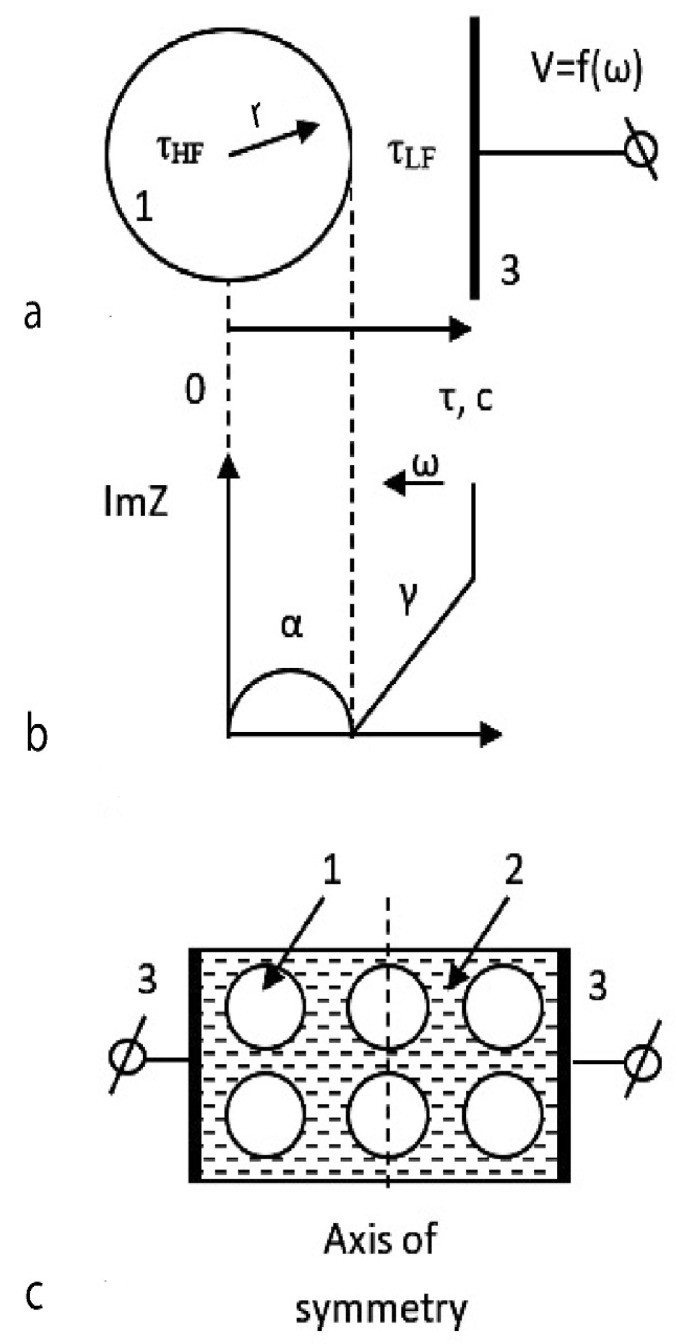
Geometric representation of a nanopowder system in projection onto the reaction coordinate axis provided that it is symmetrical concerning electrical properties (**a**); connection of spatial (r) and time (τ) coordinates with the shape of the hodograph (**b**); model representation of nanopowder system following the real spatial distribution of phases (**c**), where 1—the volume of a dielectric nanoparticle; 2—ion-conducting dispersion medium; 3—electrode r—radius vector; τ is the characteristic reaction time (time constant of structural elements).

If phases with a similar set of parameters are represented as spatial layers ordered by frequency/response time (the principle of partial linear approximation [24]), a diagram will be obtained that makes it possible to put the structure of the sample following the hodograph. Such a diagram for the object under study is shown in Figure 5a,b.

If the physical characteristics of the phases are known, then each phase can be assigned the corresponding element of the hodograph (Figure 5b). It is possible to carry out spatial modeling of the object of study under the assumption of its symmetry of electrical properties (Figure 5c), and it is possible to study electrical processes directly in the volume of each of the phases by the EIS method (Figure 5b,c).

Figure 4b shows an equivalent electrical curcuit of the measuring cell, modeled by a computer program in the form of an experimental hodograph. It can be seen that a typical hodograph can be approximated in the form of two sequentially connected parallel RC chains. Each of the RC chains has its characteristic time τ—reaction/relaxation time.

The classical RC chain characterizes a semicircle α located in the high-frequency region of the spectrum with a center shifted to the region of negative values (Figure 5b). This semicircle reflects the conductivity of structural elements with small relaxation times R1C1 = τHF. The shape of the spectrum in the form of a semicircle indicates the capacitive nature of the conductivity of the area of space where the corresponding structural elements are located.

The linear part of the low-frequency impedance indicates the predominant con-tribution of the diffusion-controlled area of the sample and characterizes the conduc-tivity of the spatial region with large intrinsic relaxation times R2C2 = τLF. This region in the impedance spectrum corresponds to a straight-line segment γ (Figure 5b).

The resulting model exactly matches the structure of the so-called Warburg, what is used in modeling the diffusion in polycrystalline solid electrolytes. We presented this diffusion impedance as a Randlis circuit, in which the Warburg element was replaced by CPE.

Thus, analysis of the shape of the spectra shown in Figure 4a allows us to assert that the sample under study is a spatially distributed heterophase system consisting of two physical phases that differ in the relaxation time of the structural elements τ and the nature of the conductivity.

For a more accurate approximation of the measuring cell, the capacitive element *C* in both circuits is replaced by a so-called constant phase element (CPE). The impedance of this element is described by the formula $Z_{CPE}\left(i\omega \right)=A^{-1}{\left(i\omega \right)}^{-n}$, where *A* is the proportionality coefficient, and n is the exponent characterizing the phase shift [24,25]. Also, the Warburg element was replaced by CPE in order to approximate the linear part of the hodograph for a heterogeneous sample with greater accuracy (Figure 4b).

### 3.2. Spatial Configuration of the System

The investigated disperse system can be schematically represented in the form of Figure 5a, assuming spatial symmetry concerning electrical properties.

At the beginning of the reaction axis, there is a circle-shaped region with a capacitive type of conductivity, which at a value of τ=1∕ω, $\omega =XcC_{1}$ goes into a region γ with a diffusion type of conductivity [26,27,28] (Figure 5a). In a real sample, only two physical phases are present, and it is known that the material of nanoparticles is a wide-gap dielectric.

With a very high probability, a part of the spectrum with a capacitive character of conductivity and small intrinsic times τ_HF_, corresponding to diffusionless polarization processes, characterizes the dielectric volume of nanoparticles α (Figure 5b). Consequently, the straight section γ (Figure 5b) with proper times τ_LF_ corresponds to the spatial region located between the particles. The boundary between the indicated regions with different types of conductivity corresponds to the interface—the surface of nanoparticles.

Thus, the structure of the sample, according to Figure 4 and the above reasoning, can be represented in the form of Figure 5c.

A phase with a capacitive character of conductivity usually corresponds to particles of a dispersed phase, and a phase with an ionic character of conductivity corresponds to a dispersion medium [29,30,31].

The geometric image of the system under study, obtained from the EIS spectra, corresponds to the simplest spatial configuration of a nanopowder dispersed system in a concentrated (compacted) form. Consequently, the EIS spectra in Figure 4 reflect the electrical properties of the phases of the sample in the form of a dispersed system.

This representation is typical for systems with liquid electrolytes, for example, for cells of lithium-ion batteries [26,27].

Table 3 shows the calculated impedance values in the bulk and on the surface of nanoparticles before and after exposure to an electric field.

The quantities of volume *σ_v_* and surface *σ_s_* conductivity were calculated by the formula:

$$\sigma_{v}=\frac{h}{R_{v}S}$$

$$\sigma_{s}=\frac{h}{R_{s}S}$$

where *h* = 4 mm is diameter, *S* = πd^2^/4 is cross-sectional area and *d* = 2 mm is height of of the sample.

It can be seen (Table 3) that exposure of the sample to a field of 5 V/mm for 10 min leads to an increase in the volume conductivity of the sample and a decrease in the surface conductivity.

Therefore, after exposure to an electric field:

− The electrical characteristics of the nanoparticle material have changed;
− The concentration of free charge carriers in the ionic environment of nanoparticles has decreased.

### 3.3. ZrO_2_ Nanoparticle as a Heterophase System

The surface is a discontinuity of the regular crystal lattice integrity. Uncompensated valence bonds are compensated for on the surface by the adsorption of molecules from the outside. In the case of oxide crystals, and especially ZrO_2_, the ionic atmosphere is mainly formed by the products of dissociative adsorption of water: H^+^ or OH^−^ ions [32,33].

**Figure 6:**
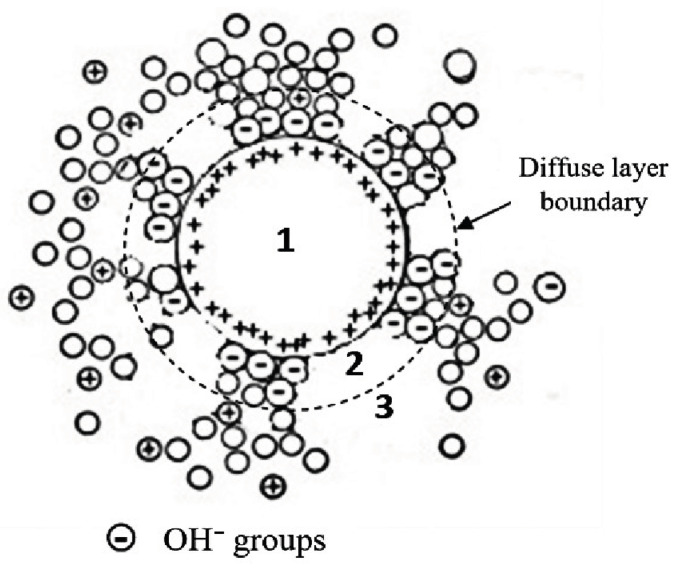
Schematic representation of a ZrO_2_ nanoparticle (1) surrounded by an ionic atmosphere, including adsorption (2) and diffuse (3) layers.

The ionic atmosphere, which is called the “hydration shell”, consists of two layers (Figure 6) [34]. The “adsorption” layer closest to the surface is bound to the surface by relatively strong (*E* > 0.3 eV [35]) chemical bonds. In the case of ZrO_2_ + 3 mol% Y_2_O_3_, the adsorption layer has a negative charge [36], it mainly contains hydroxyl groups OH^−^.

The more distant, “diffuse” layer compensates for the charge of the adsorption layer and is connected to the surface by a less strong bond, called “physical”. It mainly consists of water in molecular form.

EIS data indicate that ion atmospheres of adsorption origin transformed (condensed) during compaction, making the uniformity of electrical properties (electrical conductivity) in the space between the particles, moreover, the nature of the conductivity is diffusive.

Taking into account the fact that the volume of adsorption ion atmospheres contains products of dissociation of water molecules, it can be assumed that a change in the quantitative composition of moisture in the sample leads to a change in the concentration of free charge carriers of the diffusion type in the space between the particles. A comprehensive analysis of samples using NMR and TGA methods makes it possible to study in detail the processes occurring in the hydrate shell of nanoparticles after exposure to an electric field.

### 3.4. Results of NMR Studies

**Figure 7:**
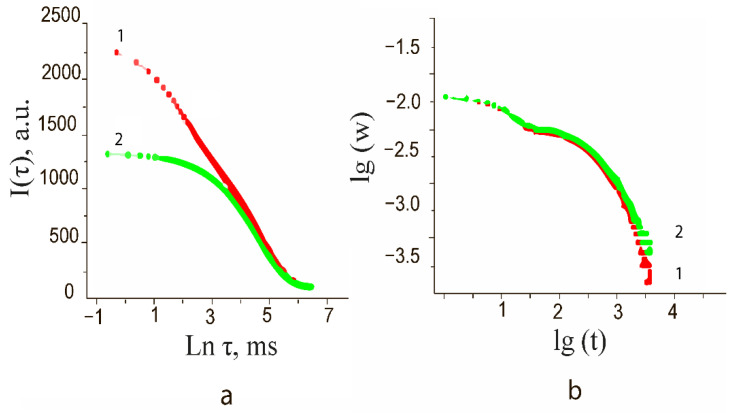
(**a**) Dependences of the amplitude of the spin-echo signal on the pulse repetition period for the control (1) and exposed to the electric field (2) samples. (**b**) Water desorption curve at 70 °C: experimental (1), and control (2).

The experimental curves obtained by NMR and TGA methods are shown in Figure 7.

The technique is based on the assumption that there are two states of water molecules in the system under study, differing in the degree of mobility. In this case, these two states can be attributed to chemically-bonded (*T*_1,*c*_) and physically-bonded sorbed water (*T*_1,*s*_).

To calculate the times *T*_1,*c*_, and *T*_1,*s*_, the relaxation curves of the magnetic moment of the hydrogen nucleus were transformed from the frequency domain to the time domain by the transformation t=1∕f. The obtained data can be approximated by Equation (3) in the form of one or two terms:

(3) $$A\left(t\right)=a\left[1-exp\left(-\frac{t}{T_{1,s}}\right)\right]+b\left[1-exp\left(-\frac{t}{T_{1,c}}\right)\right],$$

where *a*, *b* are coefficients characterizing the signal amplitude for each of the components.

The amplitude of the spin-echo signal (Figure 7a) for calculating the spin-spin relaxation time was also approximated as the sum of two terms:

(4) $$I\left(\tau \right)=a\;exp\left(-\frac{2\tau }{T_{2,\;s}}\right)+b\;exp\left(-\frac{2\tau }{T_{2,c}}\right),$$

where 2*τ* is the delay time of the spin-echo signal relative to the first RF pulse.

Calculations show (Table 4) that after exposure of the samples to an electric field *U_ex_* = 5 V/mm for 10 min, the spin-lattice relaxation time *T*_1_ increased from 88.2 to 91.2 ms.

The spin-spin relaxation time *T*_2_ after exposure decreased from 255 µs to *T*_2_ = 245 µs. The differences in values are 4%. These are relatively minor changes. However, they indicate a redistribution of the relative amount of chemically and physically bound ions on the particle surface. In particular, after exposure to an electric field in the bulk of the samples, the total content of physically bound water (spin-spin relaxation) decreases, and the proportion of chemically bound water (spin-lattice relaxation) increases.

### 3.5. Gravimetric Analysis Data

Weighing on analytical scales during the exposure time *t* = 10 min under normal physical conditions did not show changes in the mass of samples, even in the fourth sign. The mass of the samples averaged about 0.9800 ÷ 1.0200 ± 0.0001 g.

### 3.6. Thermogravimetric Analysis Data

Obtained by drying at 70 °C (desorption curves at 90 °C have a similar form) the values of the mass of the samples, converted according to (5) to the values of humidity, in logarithmic coordinates (have the form shown in Figure 7b).

(5) $$w\left(t\right)=m\left(t\right)/m_{\infty }-1,$$

where *t*—time, *m_∞_*—the mass of a dry sample.

### 3.7. Exponential Approximation–Determination of the Activation Energy of Desorption of Molecules from the Surface of Nanoparticles

The experimental desorption curves can be approximated by the kinetic moisture curve in the form of an exponent:

(6) w(t)=b·exp(−at),

Here, the coefficient *a* is related to the effective coefficient of diffusion/desorption by equation *D_eff_ = a·r*^2^, where *r*—characteristic length of a particle path during the diffusion process. Then, the value of the coefficient a has been determined, and assuming the Arrhenius dependence of the coefficient of diffusion/desorption *D_eff_* from temperature, we can calculate the activation energy by the formula [10]:

(7) $$E_{a}=R\left(\frac{T_{1}T_{2}}{T_{1}-T_{2}}\right)\ln\left(\frac{D_{1}}{D_{2}}\right),$$

where *R* is the universal gas constant.

For these samples in the range of 2.8 < *lg*(*t*) < 3.2 in double logarithmic coordinates, the kinetic desorption curve has a linear area and can be approximated by:

(8) $$\Delta m/m_{\infty }=t^{-k},$$

a scaling representation of the desorption curve is allowed [13].

The results of the analysis of functions approximating the curves (Figure 7b) in exponential and scaling representations are given in Table 5.

According to Figure 7b and Table 5 after exposure to an external electric field, the coefficients *k* of the power function, which characterizes the rate of desorption of water from the surface of nanoparticles, decreased from *k_c_* = 0.845 (for the control) to *k_exp_* = 0.786 (for the experimental) samples, indicating a slowdown in the desorption process in samples exposed to an electric field.

To calculate the activation energy *E_a_*, instead of *D*, we substitute a coefficient α with the corresponding indices in formula (7). For the control and exposed samples, the values were obtained, respectively, *E_ac_* = 1.9 kJ/mol and: *E_aexp_* = 16.0 kJ/mol. That is, the diffusion activation energy increased by 14.1 kJ/mol.

An increase (4%) in the number of chemically sorbed ions and a decrease in the rate of their desorption (7%) from the sample volume relative to the control one suggests that exposure in an electric field significantly (the activation energy of water desorption increased by a factor of 8) slows down the desorption of dissociation products of water molecules from the sample and leads to a shift in the adsorption equilibrium towards adsorption.

### 3.8. Results of the BET Method

The results of studying the specific surface of the samples by the BET method are shown in Table 6. It can be seen that the effect of an electric field on the samples is accompanied by an increase of 12% in the value of the SBET parameter (from 113.82 ± 6.83 to 128.7 ± 7.72). Taking into account that the electric field does not affect the topology of the object (Figure 3), the data of measurements by the BET method indicate an increase in the chemical activity of the surface of nanoparticles in the samples.

Thus, based on the above NMR, TGA, and BET data, in the concepts of the structure and electrical properties of the object of study proposed based on the EIS method data, it can be concluded that an external electric field leads to an increase in the energy of chemical bonding of adsorption atmosphere ions with the surface.

### 3.9. Estimated Physical Mechanism of the Effect

Classification of the effect. The samples were discharged through an ohmic load *R* = 1 MΩ, therefore, it was followed by the release of energy.

The capacitance of the system under study is significantly higher than the capacitance of a capacitor of the same geometry with metalized electrodes and a monolithic dielectric made of ZrO_2_-ceramics (*ε* = 25) $\left[C=\varepsilon \varepsilon_{0}S∕d=1.2\cdot 10^{-10}F\right]$. Consequently, the accumulation of an electric charge by the structure under study involves the bulk layers of the sample material.

There is a proportional dependence of the sample capacitance on the weight fraction of moisture. Consequently, this effect is not polarizing, however, it is caused by the transport of ion nanoparticles in an electric field [33,34,35], otherwise, an increase in the electrical conductivity of the dispersion medium at a direct current would lead to the screening of the polarization charge and a decrease in capacitance. Disperse systems based on a similar physical mechanism are considered in [37,38,39].

The effect of charge accumulation by the nanopowder system, according to NMR and TGA data, relates to the adsorption of ions of the dispersion medium by the surface of nanoparticles. At the same time, according to the EIS data, there is a change in the electrical properties of the nanoparticle material, and the value of the chemical activity according to BET increases. These empirical facts are forms of demonstration of a specific physical phenomenon associated with a change in the structure of nanoparticles at the level of the electronic subsystem of surface atoms. Taking this into account, it can be concluded that the localization of the spatially separated charge occurs on the surface of nanoparticles located in the sample volume, mainly near the electrodes [36,40] as a result of heterophase interaction.

Thus, in the functional aspect, the sample is a system of the “surface in volume” type: the functional environment is the surface located inside the volumetric object.

Next, the proposed mechanism of the effect will be considered, taking into account the above mentioned and the model concepts of the investigated object, based on the analysis of EIS spectra in the theory of contact phenomena in semiconductors [41,42,43].

### 3.10. Charge State of Nanoparticles

**Figure 8:**
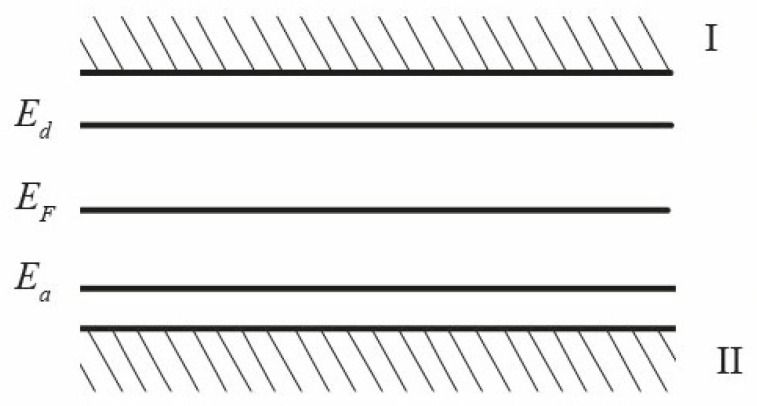
Qualitative band model of β-ZrO_2_ with yttrium (*E_a_*), with oxygen vacancies (*E_d_*). I—conduction band, II—valence band.

The mechanism of charge compensation of impurities in the bulk of dielectric nanoparticles. In a substitutional solid solution based on zirconium dioxide, the Y^3+^ impurity atoms have 1 electron less than the Zr^4+^ atoms and are acceptors, creating levels in the forbidden zone located near the top of the valence band-surface electronic eigenstates (Figure 8).

The oxygen vacancies formed for compensation of the excess volume and charge of the inovalent impurity are electron donors and create donor levels near the bottom of the conduction band (holes, Figure 8). The acceptor and donor nature of β-ZrO_2_, respectively, for Y^3+^ and oxygen vacancies, is confirmed by the results of [44].

As a result of thermal fluctuations, holes donate an electron to the conduction band (hole ionization), which, through the Coulomb interaction, finds an impurity atom and enters its orbital [45]. An impurity-vacancy dipole (IVD) of the Me–V_0_ type is formed, and the corresponding ionic bond appeared according to [37]. This is a stabilizing element of the lattice (T-phase). In this case, the impurity atom acquires a negative charge Y^3+(−1)^, and the vacancy acquires a positive charge V^(+)^. That is, donors and acceptors coexist in the form of bound charges, which are so-called impurity-vacancy dipoles of the V^(+)^-Y^3+(−)^ (IVD) type.

The concentrations of donors and acceptors are equivalent, and there is no shortage (or excess) of electrons compared to the number required for ion bonding. Therefore, in the absence of an ionic atmosphere, the material of a solid solution particle of the composition ZrO_2_-Y_2_O_3_ in the volume is electroneutral, and there is no electronic conductivity in it (it is assumed that there are no intrinsic surface electronic states [46,47]).

### 3.11. Heterophase Boundary as a Functional Heterojunction

**Figure 9:**
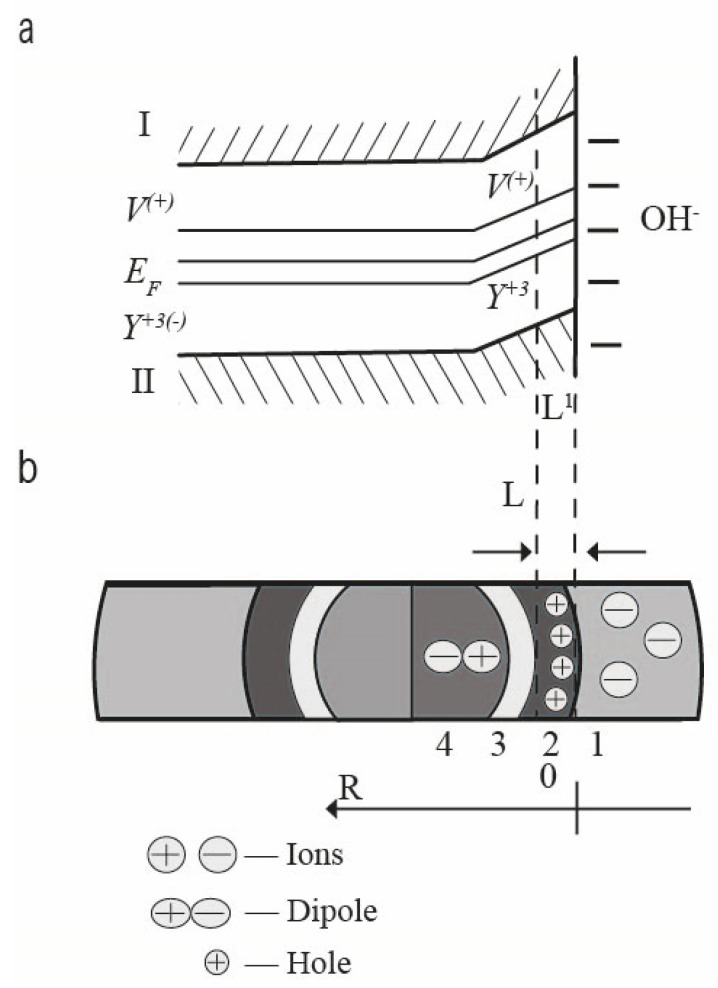
(**a**) Qualitative band model of the surface of the ZrO_2_-Y_2_O_3_ system with chemically adsorbed quasi-water molecule (OH^−^). The surface is negatively charged. I—conduction band; II—valence band. (**b**) Spatial distribution of charge in the nanoparticle volume and beyond. 1. Hydration shell. 2. Hole conductivity layer (L’). 3. Field penetration area (L). 4. Neutral dielectric core of the nanoparticle. R is the spatial coordinate.

From the standpoint of the band theory, the surface of a nanoparticle is formally a contact area of two materials with different electron work functions. The electron work function A_ZrO2_ nanoparticle is about 3.3 eV [48], and the electron work function in the case of the adsorbed molecule is equal to the ionization potential for the OH^−^ group (OH^−^ radical) according to [49] A_OH_ = I_OH_ = 13.08 eV. Therefore, when even neutral water molecules with A > A_ZrO2_ are adsorbed or approached to the surface (which is almost always done [50]), for example, H_2_O, contact physical phenomena occur, similar to those that occur when solids come into contact. In particular, the electron density of surface atoms is shifted towards the adsorption layer to a depth of *L* (Figure 9a).

In a thin layer 2 of length L’, where the bands cross the Fermi level *E_f_*, the electron passes into the conduction band *E_c_* through the tunneling effect with the probability determined by the Fermi–Dirac function [36]:

(9) $$f={\left(1+exp\left[\frac{E_{f}-E_{c}}{kT}\right]\right)}^{-1},$$

where, *k*—the Boltzmann constant; *T*—temperature.

The mechanism of adsorption within the framework of the model concepts may include:

− The destruction of the relatively weak (energy of the order of 0.1 eV [51]) bond between the Y^(+3)^- impurity and the V^(+)^ vacancy (Figure 9a) occurs in the process of adsorption;
− Localization of an electron from the crystal lattice of a nanoparticle and localization in the adsorption layer, spatial separation of the charge [52]. In this case, region 2 acquires a positive charge of the electronic type (“hole conductivity”). An electrostatic field *E_in_* is formed between the layer of ions-adsorbates and the surface, which is proportional to the difference between the work functions of A_OH_ and AZrO_2_ [53]:
  (10) $$E_{in}=\Delta A/Qd,$$
  where *Q = Σ* (*N_i_q_i_*) is the charge of the adsorption layer; Δ*A* is the difference between the work functions of the contacting materials; *N_i_*, *q_i_*—number and charge *i*—types of ions; *d* is the distance between opposite charges; *E_in_* is the internal field of the nanoparticle-adsorption atmosphere system:
  (11) $$E_{sum}=E_{ex}+E_{in},$$
  where *E_sum_* is the total field of the system; *E_ex_* is the external field of the nanoparticle-adsorption atmosphere system.

In region 3 (Figure 9b), the potential is equalized and the internal field *E_in_* is compensated. In the center, there is an electrically neutral core (4) with a dielectric character of conductivity (Figure 5a and Figure 9b).

In the presence of an adsorption atmosphere, a charge state is thermodynamically equilibrium, which implies the presence of an electron-type charge layer in the near-surface region of nanoparticles (surface impurity electronic states [50,52,54]). The surface of a nanoparticle is the interface between the opposite charge carriers of different physical nature: ions from the outside and electrons from the inside. Such structures are traditionally used as solid-state nano-ionic capacitors with ultra-high capacitance density [5].

### 3.12. Adsorption Mechanism of Charge Accumulation

**Figure 10:**
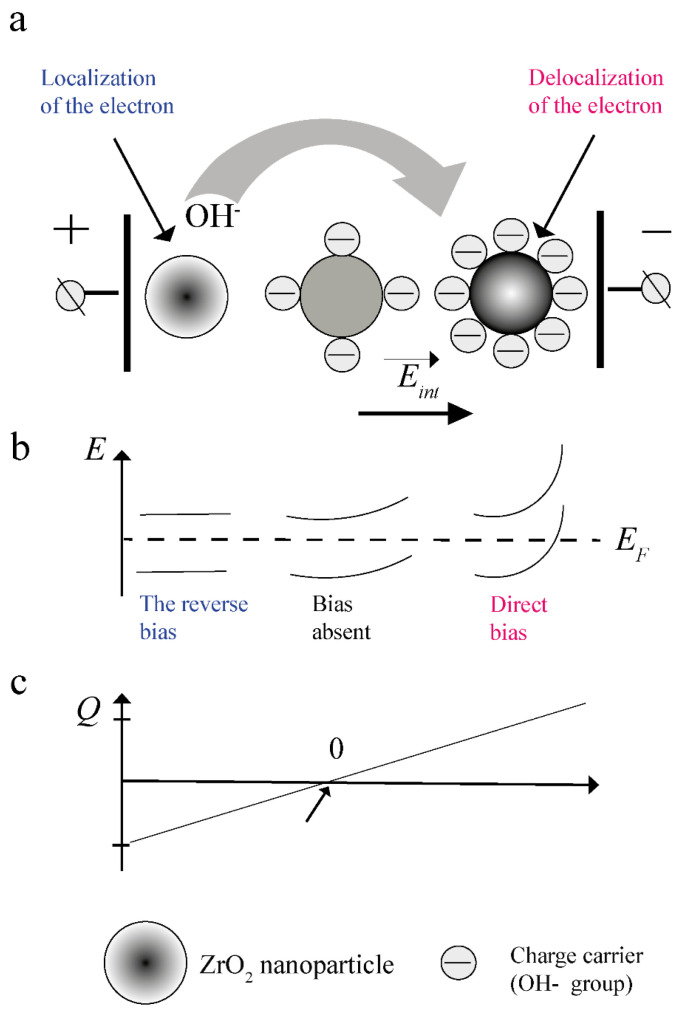
A schematic representation of the mechanism of the effect of charge accumulation by samples upon exposure to an electric field: (**a**) distribution of charge carriers along the sample height h between the electrodes after exposure to the *E_ex_* field. (**b**) The structure of the energy bands at different points of the sample in the *E_ex_* field. (**c**) Charge of the inner surface of the particle at different points of the sample in the field *E_ex_*; *E_F_* is the Fermi level.

The accumulation of charge by a dielectric nanoparticle occurs as follows (Figure 10).

The surface of the nanoparticles is electrically connected to the surface of the electrodes through ions of the dispersion medium. That is, the dispersion medium is electrically continuous and provides charge delivery to the particle surface. When the electrode potential changes, the potential on the nanoparticle surface changes. The zones bend (Figure 9a and Figure 10b) upward when a negative (direct) potential *φ-* is applied concerning the thermodynamical equilibrium potential of the nanoparticle surface *φ*_0_ and, accordingly, downward when a positive (reverse) potential *φ+* is applied. (Figure 10b).

In the first case, this leads to localization from the crystal lattice of the nanoparticle material of an additional amount of electrons in the near-surface region 2 (Figure 9 and Figure 10a). This manifests itself in the form of a decrease in the impedance of the bulk part of nanoparticles (Figure 4, Table 3) and an increase in the chemical activity of their surface (Table 6)/an increase in the activation energy of moisture desorption (Table 4 and Table 5).

In the second case, the opposite process takes place. That is, under the influence of an external electric field, in fact, the number of electrons that are potentially able to participate in the formation of a chemical bond with adsorbates decreases.

Consequently, with forwarding bias (negative potential), additional adsorption of ions will occur, and with reverse bias, desorption will occur (schematically shown in Figure 10a). According to the data of gravimetric analysis, the redistribution of the ion concentration between the electrodes will take place without the participation of the external atmosphere, presumably due to the donor–acceptor mechanism (Figure 10a). As a result, in the electric field *E_ex_*, the spatial region of the sample near the negative electrode is charged negatively, and near the positive one, respectively, positively. An internal electric field *E_int_* is created in the sample (Figure 10a,c). Proposed that these processes are equally probable, the charge of the near-surface region of the volume of nanoparticles, depending on their spatial arrangement in the volume of the sample, can be schematically represented in the form of a diagram shown in Figure 10c.

According to the BET, TGA, and NMR data, the desorption process is partially suppressed at the external fields and exposure times used in the work. Therefore, the spatial distribution of oppositely charged regions in the sample volume is probably not symmetric, as in Figure 10c.

### 3.13. Clarification of the Adsorption Mechanism–Assessment of the Capacitive Characteristics and Efficiency of the System under Study as an Electric Energy Storage Device

Quantitative estimates of the fraction of effective charge carriers in the system and the effective area of the heterojunction were made to determine the mechanism of charge separation.

The number of effective charge carriers in the system can be determined by knowing their total number, the capacitance of an elementary element, and the total capacitance of the system.

### 3.14. Determination of the Total Number of Charge Carriers in the System

Let the weight fraction of moisture in the sample Δ*m* = 3.2 wt%; sample mass *m_sample_* = 1 g, then, the mass of water in the sample *m_water_* = 0.032 g.

Molar mass of the OH-group: *M_OH_ = M*_0_ *+ M_H_*, where

*M*_0_ = 16 at. units—molar mass of oxygen;

*M_H_* = 1 at. units—molar mass of hydrogen;

*M_OH_* = 16 + 1 = 17 at. units;

Moles in *m_wate_*_r_ = 0.032 g of water:

(12) $$N_{3,2}=m_{water}∕M_{OH}$$

*N*_3,2_ = 0.032/17 = 0.0019 mol.

The number of molecules

(13) $$N_{m}=N_{A}\cdot N$$

where

*N_A_* = 6.02 × 10^23^ units/mol—Avogadro’s number.

At Δ*m* = 3.2%, the sample will contain:

*N_m_*_3,2_ = 6.02 × 10^23^ × 0.0019 = 1.14 × 10^21^ units of OH—molecules.

### 3.15. Determination of the Total Number of Charge Carriers in the System

According to the adsorption model (Figure 9), an elementary capacitive element can be considered a conditional dipole induced at a heterojunction during the adsorption of a carrier using a shift in the electron density. An induced dipole can be represented as two conductors with a potential difference of *V* or as an ideal capacitor. In this case, the capacitance [5,53] will correspond to it:

(14) $$C_{OH}=q/V,$$

where

*q* is the charge of one of the conductors.

In electrochemical processes accompanied by the transfer of charged particles across the interface, a potential difference, called the electrode potential *E_φ_*, arises [55]. For the interaction of ZrO_2_ with hydrogen, this value is known: *EφZrO_2_* − H = −1.43–−1.55 V [56].

In our case, at zero external potential (bias voltage), this electrode potential will correspond to the contact potential difference *Vk*.

*EφZrO_2_* − H = −1.43–−1.55 V

*q* = *e* = 1.6 × 10^−19^ C [1]

*C_OH_* = 1.6 × 10^−19^ C/1.5 V ≈ 1 × 10^−19^ F.

### 3.16. Determination of the Degree of Use of the Heterojunction Surface

The slot for the OH-group, according to [57,58], is about *S_OH_* = 10.2 Å = 10.2 × 10^−20^ m^2^. The area occupied by molecules *S_OHsum_ = S_OH_N_m_*. For Δ*m* = 3.2%,

*S_OHsum_* = 10.2 × 10^−20^ m^2^ × 1.14 × 10^21^ = 116 m^2^.

That is, at Δ*m* = 3.2 wt%, the surface is filled by the amount

(15) $$k=\frac{S_{OHsum}}{S_{BET}}\cdot 100\%,$$

*k* = (116/113.8) × 100% = 101%.

That is, all but with a few exceptions, the molecules present in the system are located on the surface of the heterojunction.

Consequently, for this system, charge and adsorption equilibria under normal conditions (there is no external field) take place when the adsorption layer is filled. However, it also follows from this that the adsorption of charge carriers in an electric field cannot occur directly on the surface of the heterojunction, as was assumed in the adsorption model in Figure 9 and Figure 10.

### 3.17. What Is the Mechanism for the Shift of the Electron Density from the Bulk of Nanoparticles to the Adsorption Layer in an Electric Field?

According to the adsorption mechanism of charge accumulation (Figure 9 and Figure 10), the primary in the interaction of a nanopowder system with an electric field is the generation of nonequilibrium electron-type charges in the near-surface region of the material of nanoparticles. The energy stimulus for this is a change in the contact potential difference and the height of the energy barrier. To compensate for this charge, according to the model (Figure 9 and Figure 10), water molecules are adsorbed on the surface of the heterojunction. However, quantitative estimates show that the adsorption of water directly on the surface of nanoparticles cannot occur, because it is already filled in an equilibrium state.

Consequently, charge accumulation can be realized either as a result of the transition of a certain amount of adsorbates from the physical to the chemical form of adsorption (the binding energy is of the order of 105–194 kJ/mol [59]) (1) or as a result of the movement of a part of the physical adsorption molecules from the oppositely charged pole of the sample (2). The influx of ions from the outside is not considered, since no changes in the mass of the samples were recorded during exposure to an electric field.

The possibility of realizing the first mechanism is evidenced by the NMR data, according to which, after exposure to an electric field in the sample, the number of adsorbed molecules is redistributed towards chemical adsorption.

The second assumption is supported by the relatively small (compared to the binding energy in the adsorption layer) value of the specific energy of thermal desorption of molecules in the samples exposed to the electric field: *E_aexp_* = 16.0 kJ/mol (TGA data, Table 5). Thus, both mechanisms are likely to take place. However, from comparing the magnitude of the effect on NMR and TGA, it can be assumed that the second mechanism is more probable.

The role of physical adsorption can also be estimated using data on the capacitance of the system at degrees of moisture greater than η = 35% (Δ*m* = 3.2%).

### 3.18. Determination of the Number of Conditional Dipoles in the Generalized Heterojunction

According to the above reasoning and [5], the elementary capacitive element in the system under consideration is the conditional dipole (14).

By definition, a conditional dipole corresponds to a charge equal in magnitude to the charge of one electron *e* = 1.6 × 10^−19^ C, and the capacitance *C_dip_* = 1 × 10^−19^ F. Knowing the total capacitance of the system and the moisture content in it, it is possible to estimate the total number of charge carriers in the adsorption layer (12)–(13) and the number of conditional dipoles on the generalized surface of the heterojunction *N_Cdip_* (16). For example, for samples with moisture content in the amount of Δ*m* = 3.2%, according to Table 2, for the samples’ “charging” modes used in the work (5 V/mm, 10 min), the specific electrical capacitance of the surface is *C* = 58.95 μF. The number of dipoles *N_Cdip_*_3,2_ is found as [5]:

(16) $$N_{Cdip}=\frac{C}{C_{dip}}\;,$$

(17) $$N_{Cdip3,2}=\frac{58.95\times 10^{-6}}{1\times 10^{-19}}=6.5\times 10^{13},$$

The calculated values of *N_Cdip_* and *N_m_* are given in Table 7.

As can be seen from Table 7, when the sample is saturated with moisture, the fraction of “effective” charge carriers increases. In particular, for Δ*m* = 1.7 and 6.6 wt%, this difference is 8 times. Provided that the quantitative composition of the ion layer located near the surface does not change, this indicates a significant effect of physical adsorption ions on the capacitance of the system. Additional adsorption of the OH^−^ groups physically connected to the surface leads to the compensation of the excess electron charge and significantly increases the concentration of conditional dipoles.

Thus, according to the NMR and TGA data, it can be concluded that, in an electric field, the compensation of the excess electronic charge formed on the inner side of the nanoparticle surface under the action of an external electric field can occur according to two mechanisms. The first mechanism is an increase in the energy of the chemical bond of functional groups localized on the surface. According to the second mechanism, the compensation of the excess electronic charge induced by the electric field in the near-surface layer of nanoparticles occurs through the superposition of fields of a certain amount of molecules relatively weakly bound to the surface of molecules-adsorbates (OH^−^ groups). The number of OH^−^ groups per one electron delocalized from the crystal lattice of the nanoparticle material by an electric field can be determined using the BET data.

### 3.19. Determination of the Ratio between the Number of Electrons and Adsorbates Delocalized from the Material Volume of Nanoparticles

Assuming that the increase in the specific surface area of the samples by Δ*S* = 14.9 m^2^/g (Table 6) at (Δ*m* = 3.2%), indicated by the BET method, is due to a relatively long-term increase in the chemical activity of the nanoparticle surface as a result of localization by the electric field of the excess electron charge, and if the contribution of the “first” mechanism of its compensation is neglected, the corresponding equivalent number of physically bound OH^−^ groups in the adsorption layer *N_COH_* can be estimated:

(18) $$Nc_{OH\;3,2}=\frac{\Delta S}{S_{OH}},$$

(19) $$Nc_{OH\;3,2}=\frac{14.9\frac{m^{2}}{g}}{10.2\times 10^{-20}m^{2}}=1.46\times 10^{20},$$

If the increase in *S_BET_* is due solely to the induction of electrons, then for one electron, there is:

(20) $$n=\frac{Nc_{OH\;3,2}}{N_{Cdip}},$$

(21) $$n_{\;3,2}=\frac{\;1.46\times 10^{20}}{6.5\times 10^{13}}=2.2\times 10^{6},$$

This means that more than two million OH^−^ groups are needed to compensate for one electron and accumulate the system on a generalized heterojunction of a charge with a value of *C_dip_* = 1 × 10^−19^ F.

### 3.20. Evaluation of the Efficiency of Interaction of OH^−^ Groups with the Surface of a Generalized Heterojunction

Consequently, the reason for the low efficiency of charge accumulation by the nanopowder system is due to the poor ion transport properties of the dispersion medium.

Hydrated moisture from the atmosphere is not an optimal electrolyte; however, using the capabilities of the surface of nanoparticles, even with such negligible efficiency, gives relatively tangible results.

Table 7, in terms of 1 g, shows the values of the capacitance of the system with different amounts of adsorbed moisture, provided that all molecules interact effectively with the surface of the nanoparticle:

(22) $$C_{max}=C_{dip}\cdot N_{m},$$

The maximum capacitance of a system based on a wide-gap dielectric ZrO_2_ with a relative fraction of moisture in the sample Δ*m* = 6.6% (in terms of 1 g) will be: 

*C_max_* 6.6 = 1 × 10^−19^ × 2.3 × 10^21^ = 0.23 × 10^3^ F = 230 F. This capacitance is relatively high, even for advanced liquid electrolyte “supercapacitor” designs.

It can be seen that the charge accumulation efficiency (*C/C_max_*) of 100%, based on the experimental data (Table 7), is proportional to the concentration of charge carriers. With an increase in the proportion of moisture from 3.2 to 6.6% by weight, the efficiency of charge accumulation increases by almost 14 times.

Thus, there is reason to believe that due to the high dielectric constant of ZrO_2_ in the bulk of nanoparticles, it is possible to achieve a high energy density, but the hydration shell is ineffective due to the low concentration of free charge carriers-ions. The above results and reasoning give reason to believe that when optimizing the heterophase interaction, for example, by selecting a dispersion medium on a generalized functional heterojunction based on nanoparticles of the solid solution under study with a diameter of 7.5 nm, it is possible to obtain a capacitance density of at least 230 F/g.

The sample can be in the form of 3D and 2D objects and scaled up to one nanoparticle. The use of a non-hygroscopic ion-exchange dispersion medium will ensure the stabilization of the electrical parameters of the charge accumulators, otherwise, it can be effectively used as humidity sensors.

The use of nanoparticles of a wide-gap dielectric ZrO_2_ as a functional element of pulsed energy storage has several advantages over carbon analogs. These include the possibility of creating solid-state devices with a relatively high operating temperature (the latter is limited by the onset of thermal diffusion processes in the bulk of nanoparticles (T_D_ ≈ 600 °C)) and the possibility of scaling into the nanoscale range by up to one particle.

## 4. Conclusions

1. It has been determined, that nanoparticles of a solid solution based on ZrO_2_ with a size less than 100 nm, in a compact compacted with high hydrostatic pressure (500 MPa), in the form of a tablet with a diameter of 18 and a height of 2 mm are capable of accumulating an electric charge in an electric field *E* = 5 V/mm under normal external physical conditions (atmospheric pressure *P* = 762 Hg, humidity η in the range 25–85% and temperature *T* = 18 °C). The maximum value of the specific electrical capacitance *C* ≈ 1.87 × 10^−4^ F/g at direct current was established for samples containing 6.6 wt% moisture with a particle size of *d* = 7.5 nm.

2. It has been determined that the effect of the accumulation of an electric charge by a nanopowder oxide system has a dimensional character. Notably, it was shown that the capacitance of samples with a nanoparticle size of 7.5 nm exceeds the capacitance of samples with a particle size of 96 nm by more than 10 times.

3. It was found that the value of the sample capacitance depends significantly on the content of adsorbed moisture in it. In particular, it was shown that the capacitance of a sample with a mass fraction of moisture of 6.6% is 30 times higher than the capacitance of a sample, with a mass fraction of moisture of 1.7% (practically dehydrated).

4. It was found that the effect of an electric field on compacts is accompanied by a 13.09% increase in the specific surface area *S_BET_* (from 113.82 ± 6.83 to 128.7 ± 7.72), an increase in the activation energy of water desorption from the surface of nanoparticles *E_a_* by 14.1 kJ/mol (from 1.9 to 16.0 kJ/mol), with a simultaneous decrease in the diffusion rate *k* by 7% and by 4% in the spin-spin relaxation time (*T*_2_ = from 255 μs to 245 μs).

5. The adsorption mechanism of a certain effect is proposed within the framework of the dispersed media model. According to this model, nanopowder is considered as a two-phase system in which nanoparticles of a solid solution based on ZrO_2_ are a dispersed phase, and the surrounding gaseous ionic atmosphere is a dispersion medium.

6. Based on the EIS data, and taking into account the assumption of the symmetry of the system concerning the electrical properties, it has been determined that the dispersion medium (ionic atmosphere) is electrically continuous and has an ionic type of conductivity. It is concluded that the surface of the nanoparticles is electrically associated with the electrode.

7. From the standpoint of the theory of contact phenomena in semiconductors, the heterophase interaction between the nanoparticles and the external ionic atmosphere is considered.

7.1. It was shown that the interface is a bulk functional heterojunction, which is essentially similar to a semiconductor one in physical processes.

7.2. It was defined that the accumulation of an electric charge by the system is a consequence of the redistribution of the electron density between the crystal lattice of the nanoparticle material and the sorbed molecule due to the difference in the work function of the electrons from these materials. It was shown that it is the adsorption of OH^−^ ions that lead to the confinement of an electric charge in the system in the form of so-called “conditional dipoles”—excitons localized at the interface between the nanoparticle material and the gas phase.

Based on the NMR and BET data, quantitative estimates were made of the total number of charge carriers in the adsorption layer and the degree of surface coverage with adsorbates. It was shown that under normal conditions (η = 35%), the surface of nanoparticles (7.5 nm) in the sample is completely (101%) covered with water molecules and is in a state of adsorption and charge equilibrium.

Based on NMR and TGA data using a conditional dipole model as an elementary capacitive cell of the structure, it was shown that the compensation of the electric field-induced electronic charge from the side of the nanoparticle material occurs in two ways:

− Through the transition of a part of physically sorbed molecules into a state with a stronger chemical bond (1);
− By superposition of the fields of physically bound molecules of the adsorption layer (2). It was shown that the second mechanism has a significantly higher order of realization probability.

It was determined that the interfacial interaction between both components of the bulk heterojunction has relatively low efficiency. In particular, it was found that to compensate in the crystal lattice of the nanoparticle material for an electric charge equal to the charge of one electron, more than 2 × 10^6^ charge carriers are needed from the side of the adsorption layer.

It was found that the efficiency of interfacial interaction can be increased by 30 times by increasing the proportion of hydration moisture Δ*m* from 3.2 to 6.6 wt%. It is assumed that replacing OH^−^ groups with ions with a higher electrical activity will significantly increase the efficiency of a storage cycle based on the considered bulk heterojunction.

When optimally selecting a dispersion medium on particles of a solid solution under study with a diameter of 7.5 nm, it is possible to obtain a capacitance density of at least 230 F/g.

It is concluded that the use of nanoparticles of a wide-gap dielectric ZrO_2_ has many advantages over carbon analogs. Among them, is the possibility of creating solid-state devices with an operating temperature of more than 300 °C and the ability to scale up to one nanoparticle.

Using the results presented in the paper, we can assume that the functional use of the generalized surface of ZrO_2_ nanoparticles may be able to solve the old technological problem of tunnel leakage currents that arose during the development of solid-state submicroscopic electric energy storage devices for nano and microsystem technology [5].

## Figures and Tables

**Table 1:** Calculated data for the capacitance of samples with different particle sizes measured at atmospheric air humidity η = 85%.

*d*, nm	*U*_0_, V	*U*, V	*t_dis_*, s	*C*, μF	*a*	*k*
7.5	1.72	0.66	180	187.8	1.36	0.317
9	1.36	0.468	150	140.22	1.11	0.214
12	0.98	0.374	150	155.05	0.667	0.288
18	0.78	0.325	60	68.43	0.667	0.0846
23	0.56	0.214	60	62.25	0.462	0.0994
50	0.22	0.082	25	24.98	0.243	0.0270
67	0.09	0.033	20	19.93	0.108	0.0116
96	0.017	0.006	20	19.2	0.0234	0.00384

**Table 2:** The electric capacitance of a sample with a particle size *d* = 7.5 nm at direct current at different moisture content.

Δ*m*, Weight %	*U*_0_, V	*U*, V	*t_dis_*, s	*C*, μF
0	1	0.4	5	5.45
1.7	3.3	0.96	6	6.12
2.2	3.42	1.16	7	6.47
2.9	2.6	1.02	20	21.37
3.2 ^1^	2.49	0.9	60	58.95
4.9	2.35	0.89	90	92.69
5.5	2.35	0.87	150	150.95
6.6	1.72	0.66	180	187.84

^1^ Corresponds to atmospheric humidity η = 35% at normal pressure.

**Table 3:** Calculated values of the volume (*R_v_*) and surface (*R_S_*) parts of the impedance of nanoparticles before and after exposure to an electric field.

	*R_v_*, Ohm	*σ_v_*, [Ohm·m]^−1^	*R_S_* Ohm	*σ_S_*, [Ohm·m]^−1^
Control	6.3 × 10^5^	2.2 × 10^−3^	2.6 × 10^4^	0.049
Experiment	4.1 × 10^4^	3.1 × 10^−2^	5 × 10^6^	2.5 × 10^−4^

**Table 4:** Results of analysis of NMR curves.

	*T*_1_, 10^−3^ s	*T*_2_, 10^−6^ s
Control	88.2	255
Experiment	91.2	245

**Table 5:** Values of the coefficients α_70°C_, α_90°C_, obtained after approximating the curves (Figure 7b) according to Formula (6), as well as the coefficients of the desorption rate *k*_70__°__C_, *k*_90__°__C_, calculated from the power-law dependence of weight loss on time for control (CONTROL) and exposed to an electric field (EXPERIMENT) samples.

	α_70°C_, s^−1^/*t*, s	α_90°C_, s^−1^/*t*, s	*k*_70°C_/g(*t*)	*k*_90°C_/g(*t*)
Control	6.45·10^−4^/(1900–2400)	6.69·10^−4^/(1000–2100)	0.845/(2.8–3.2)	0.818/(2.8–3.2)
Experiment	5.64·10^−4^/(1800–2600)	7.69·10^−4^/(1000–2200)	0.786/(2.8–3.2)	0.735/(2.8–3.2)

Where: α_70°__C_, α_90°__C_—desorption rates at 70 and 90 °C; *k*_70°__C_, *k*_90°__C_—coefficients of desorption rate at 70 and 90 °C.

**Table 6:** Measurement data of the specific surface of the samples with *d* = 7.5 nm by BET before and after exposure to an electric field.

Type of Samples ^1^	Mass of Dry Powder, g	*S_BET_*_,_ m^2^/g	SKO
Control	0.2236	113.8 ± 6.8	0.0145
After exposure ^2^	0.2147	128.7 ± 7.7	0.0088

^1^ Sample: ZrO_2_—3 mol. %Y_2_O_3_, 400 °C, 500 MPa; ^2^ E-field exposition: 5 V/mm, 10 min, η = 35%.

**Table 7:** Calculated values of charge carriers and sample capacities for different weight fractions of moisture.

Δ*m*, %	*C* 10^−6^, F	*N_m_* 10^21^, Units	*N_Cdip_* 10^13^*,* Units	*(N_Cdip_/N_m/_)* 10^−6^, %	*C_max_*, F	*C/C_max_* 10^−6^, %
1.7	6.12	0.60	0.68	1.13	110	5.56
2.2	6.47	0.78	0.72	0.92	110	5.88
2.9	21.37	1.38	2.37	1.72	138	15.48
3.2 ^1^	58.95	1.14	5.70	5.74	151	39.03
4.9	92.69	1.75	10.30	5.88	175	52.95
5.5	150.95	1.93	16.77	8.69	193	78.24
6.6	187.84	2.30	20.87	9.07	230	81.72

^1^ Corresponds to atmospheric humidity η = 35%. *m* = 1 g. *N_m_* is the number of molecules in the sample. *N_Cdip_*—the number of molecules in the sample participating in the accumulation of charge (the number of conditional dipoles). *C_max_* is the maximum possible capacitance based on the total amount of OH^−^ ions.

## Data Availability

Not applicable.
